# Cluster of Legionnaires’ Disease in an Italian Prison

**DOI:** 10.3390/ijerph16112062

**Published:** 2019-06-11

**Authors:** Teresa Fasciana, Chiara Mascarella, Salvatore Antonino Distefano, Cinzia Calà, Giuseppina Capra, Angela Rampulla, Paola Di Carlo, Mario Palermo, Anna Giammanco

**Affiliations:** 1 Department of Health Promotion, Mother and Child Care, Internal Medicine and Medical Specialties, University of Palermo, 90133 Palermo, Italy; chiaramascarella@hotmail.it (C.M.); salvatore.distefano@unipa.it (S.A.D.); cinzia.cala@unipa.it (C.C.); giuseppina.capra@unipa.it (G.C.); paola.dicarlo@unipa.it (P.D.C.); anna.giammanco@unipa.it (A.G.); 2 Unit of Microbiology, Virology and Parasitology, A.O.U.P, 90127 Palermo, Italy; 3 Laboratory of Public Health, ASP 6, 90141 Palermo, Italy; a.rampulla@yahoo.it; 4 Sicilian Health Department, Public Health and Environmental Risks Service, 90127 Palermo, Italy; mario.palermo@regione.sicilia.it

**Keywords:** *Legionella pneumophila*, prison, water, systems, cluster

## Abstract

Background: *Legionella pneumophila* (Lp) is the most common etiologic agent causing Legionnaires’ Disease (LD). Water systems offer the best growth conditions for Lp and support its spread by producing aerosols. From 2015 to 2017, the Regional Reference Laboratory of Clinical and Environmental Surveillance of Legionellosis of Palermo monitored the presence of Lp in nine prisons in Western Sicily. During this investigation, we compared Lp isolates from environmental samples in a prison located in Palermo with isolates from two prisoners in the same prison. Methods: We collected 93 water samples from nine Sicilian prisons and the bronchoalveolar lavages (BALs) of two prisoners considered cases of LD. These samples were processed following the procedures described in the Italian Guidelines for the Prevention and Control of Legionellosis of 2015. Then, genotyping was performed on 19 Lp colonies (17 from water samples and 2 from clinical samples) using the Sequence-Based Typing (SBT) method, according to European Study Group for Legionella Infections (ESGLI) protocols. Results: Lp serogroup (sg) 6 was the most prevalent serogroup isolated from the prisons analyzed (40%), followed by Lp sg 1 (16%). Most of all, in four penitentiary institutions, we detected a high concentration of Lp >10^4^ Colony Forming Unit/Liter (CFU/L). The environmental molecular investigation found the following Sequence Types (STs) in Lp sg 6: ST 93, ST 292, ST 461, ST 728, ST 1317 and ST 1362, while most of the isolates in sg 1 belonged to ST 1. We also found a new ST that has since been assigned the number 2451 in the ESGLI-SBT database. From the several Lp sg 1 colonies isolated from the two BALs, we identified ST 2451. Conclusions: In this article, we described the results obtained from environmental and epidemiological investigations of Lp isolated from prisons in Western Sicily. Furthermore, we reported the first cluster of Legionnaires’ in an Italian prison and the molecular typing of Lp sg 1 from one prison’s water system and two BALs, identified the source of the contamination, and discovered a new ST.

## 1. Introduction

*Legionella pneumophila* (Lp) is a Gram-negative water-borne pathogen that causes a severe form of pneumonia known as Legionnaires’ Disease (LD) [1]. It can create a public health problem when it grows and spreads in hot water systems, cooling towers, spa pools, dental unit waterlines, etc. [2].

Recently, the need to reinforce epidemiological surveillance programs, increase diagnostic techniques and set up preventive measures—e.g., searching for causes of infection, periodic checks of drinking water supply systems, and installation of effective disinfection systems—has become a priority concern [3].

The Italian Surveillance System dictated that physicians must fill in a form for every case of LD and send it through Local Health Units to the National Institute of Health (Istituto Superiore di Sanità, ISS). Thus, LD is a notifiable disease [4].

The European Study Group for Legionella Infections (ESGLI) established Sequence Based Typing (SBT) as a method of use to compare clinical and environmental Lp isolates worldwide and to investigate the distribution of these strains in specific areas [5,6].

The number of cases of LD has increased gradually over the years, both in Europe and in Italy, in the latter due mainly to the numerous receptive structures (hotels, spas, etc.) [7]. In 2015, most cases in Italy were community acquired (78.8%), followed by travel-associated (12.7%) and healthcare-associated (5.3%) cases. Particularly, in Sicily, 143 total cases were reported in the last fourteen years (2004–2017) [8].

Until now, in Sicily, the presence of Lp was monitored only in the water systems of hotels, hospitals, beach showers, cruise ships and ferries [9,10,11].

In 2015, after the approbation of a research project, we put our attention on prison water system surveillance. The aim of the project was to evaluate the risk for immunocompromised hosts in a high risk population of contracting LD.

Here, we present the first Italian study to monitor and molecular type Lp in water from prison water systems in Western Sicily. During the study period, the first cluster was from a prison in Palermo.

## 2. Materials and Methods

### 2.1. Environmental Sampling before the Cluster

Four prisons located in Palermo (1PA, 2PA, 3PA, 4PA), three in Trapani (5TP, 6TP, 7TP) and two in Agrigento (8AG, 9AG), all cities in Sicily, detaining in total around 3104 prisoners, were included in the surveillance study. A total of 93 water samples were collected from June 2015 to October 2017. Samples of both hot and cold water were taken.

The principal sites of sampling were as follows: the municipal water storage tank, the sinks, the boilers, and the showers on every floor in each prison.

Water contamination was monitored only for Legionella bacteria, following the procedure reported in the Italian Guidelines for the Prevention and Control of Legionellosis (2015) [4]. The 1 L water samples were filtered through sterile 0.22 μm filter membranes (Millipore, Bedford, MA, USA) using a vacuum pump. After filtration, membranes were aseptically removed, placed in sterile, screw-capped containers with 10 mL of the original filtrates, and vigorously vortex-mixed for 5 min. Half of each sample was placed in a 50 °C water bath for 30 min to reduce contamination by thermosensitive microorganisms. Aliquots of 0.1 mL of the samples (with and without heat pre-treatment) were spread over the BCYE Agar (Buffered Charcoal Yeast Extract Becton, Dickinson, Franklin Lakes, New Jersey, USA) and incubated at 36 °C for 10 days in a humid environment with CO_2_ at 2.5%.

The suspected colonies were subcultured on BCYE Agar without Cysteine, with Pimaricin (Becton, Dickinson, Franklin Lakes, New Jersey, USA) and BCYE Agar. Only the colonies grown on BCYE Agar were regarded as the *Legionella* genus.

### 2.2. Clinical and Environmental Sampling during the Cluster

Clinical samples, bronchoalveolar lavages (BALs), were obtained from two prisoners detained in one of four prisons in Palermo (1PA). The two prisoners were held in different cells on the same floor of the prison and were both hospitalized with symptoms of lower respiratory tract infection.

The BAL samples were decontaminated by heat treatment (50 °C for 30 min), plated on BCYE Agar, and incubated at 36 °C for 10 days in a humid environment with CO_2_ at 2.5%.

As well as BAL samples, urine samples were collected to determine the presence of soluble Lp sg 1 antigen, using rapid immunochromatographic (ICT) assay (BinaxNOW^®^ Legionella Urinary Antigen, Alere, Milan, Italy). The first case of LD occurred on 25 May 2017 and a new case appeared in 1PA three months later (26 August 2017).

This prison held about 1.270 prisoners.

During the first and second cases of LD, we conducted other environmental investigations of Lp sg 1 in prison 1PA; we therefore reinvestigated the hot and cold water system of 1PA which had been included in the initial surveillance project and had not produced positive results for Lp sg 1.

### 2.3. Serological Identification of Legionella

Colonies grown on BCYE Agar were identified using a latex agglutination test with polyvalent antisera (Oxoid Spa, Milan, Italy). A total of 47 Lp colonies from water samples and 10 from clinical samples were frozen at −80 °C.

### 2.4. Molecular Investigation

A molecular study was conducted on 19 Lp colonies: 17 from water samples and 2 from clinical samples. Genotyping was performed using the SBT method, following ESGLI protocols and using seven genes (*flaA, pilE, asd, mip, mompS, proA,* and *neuA*) [12,13].

For non-sg 1 Lp strains from which *neuA* could not be amplified, primers targeting *neuAh* were used, as suggested by the ESGLI [14].

The allelic profile that determined Sequence Type (ST) was defined by assigning a distinct allele number to each gene using the ESGLI–SBT database [15].

## 3. Results

### 3.1. Environmental Evaluation before the Cluster

Of the 93 water samples, 49 (52.7%) were negative for the presence of Lp, and 44 (47,3%) were positive. The negativity was reported as Lp <100 CFU/L according to the Italian Guidelines. Among the positive samples, Lp sg 6 was the prevalent serogroup (40%) compared to Lp sg 1 (16%) (Table 1).

All nine prisons showed at least one positive sampling point for Lp sg 1 and/or Lp sg 6, as shown in Table 1. In prisons 1PA, 2PA, 5TP and 6TP, we found only Lp sg 6. In prison 9AG, we detected only Lp sg 1. Lp sg 1 was isolated from three cold water samples and six hot water samples from prisons 3PA and 4PA, from two cold and two hot water samples from prisons 8AG and 9AG, and from no cold water samples and no hot water samples from prisons 5TP and 6TP.

The levels of Lp sg 1 and sg 6 before the cluster are shown in Table 1, and the different concentrations of Lp sg 1 and sg 6 in hot and cold water are reported in Table 2.

Regarding the molecular investigation, only ST 1 was identified in Lp sg 1, while in sg 6, the following STs were found: ST 93, ST 292, ST 461, ST 728, ST 1317 and ST 1362.

### 3.2. Clinical and Environmental Evaluation during the Cluster

Initially, a quicker more basic procedure was conducted on urine samples from two prisoners by a different hospital. These samples tested positive for Lp sg 1 antigen. Consequently, the BAL samples were sent to our hospital in order to obtain the isolates. Colonies were identified by latex agglutination (Oxoid Spa, Milan, Italy) as Lp sg 1. The molecular study demonstrated that the allelic profile 2, 10, 3, 1, 9, 1, 20, is a new ST that has since been assigned the number 2451 in the ESGLI- SBT database.

Regarding the environmental evaluation in the water system of prison 1PA, on 29 May 2017, state prison system facilities management staff collected hot and cold water samples from the municipal water storage tanks (n = 2), the boilers (n = 4), the showers (n = 2) located on the floor where the prisoner with the first confirmed case was detained, and the sinks (n = 2) in his cell. Lp sg 1 was detected only in the storage tank (10^2^ CFU/L) and the typing investigation demonstrated that the water isolate had the same ST profile as the clinical strain.

On 28 August 2017, after the second case presented, water samples were collected from the municipal water storage tanks (n = 2), the boilers (n = 4), and the showers (n = 2) located on the floor where the prisoner with the second confirmed case was detained, as well as from the sinks (n = 2) in his cell. Lp sg 1 was detected in the storage tanks (10^2^ CFU/L) and in hot and cold water from the showers (10^2^ CFU/L), and it was the same ST as the clinical strain. The other samplings of hot and cold water were <100 CFU/L. After this cluster occurred, the water distribution system was cleaned following the Italian Guidelines and all the samples resulted negative for Lp [4].

**Figure 1:**
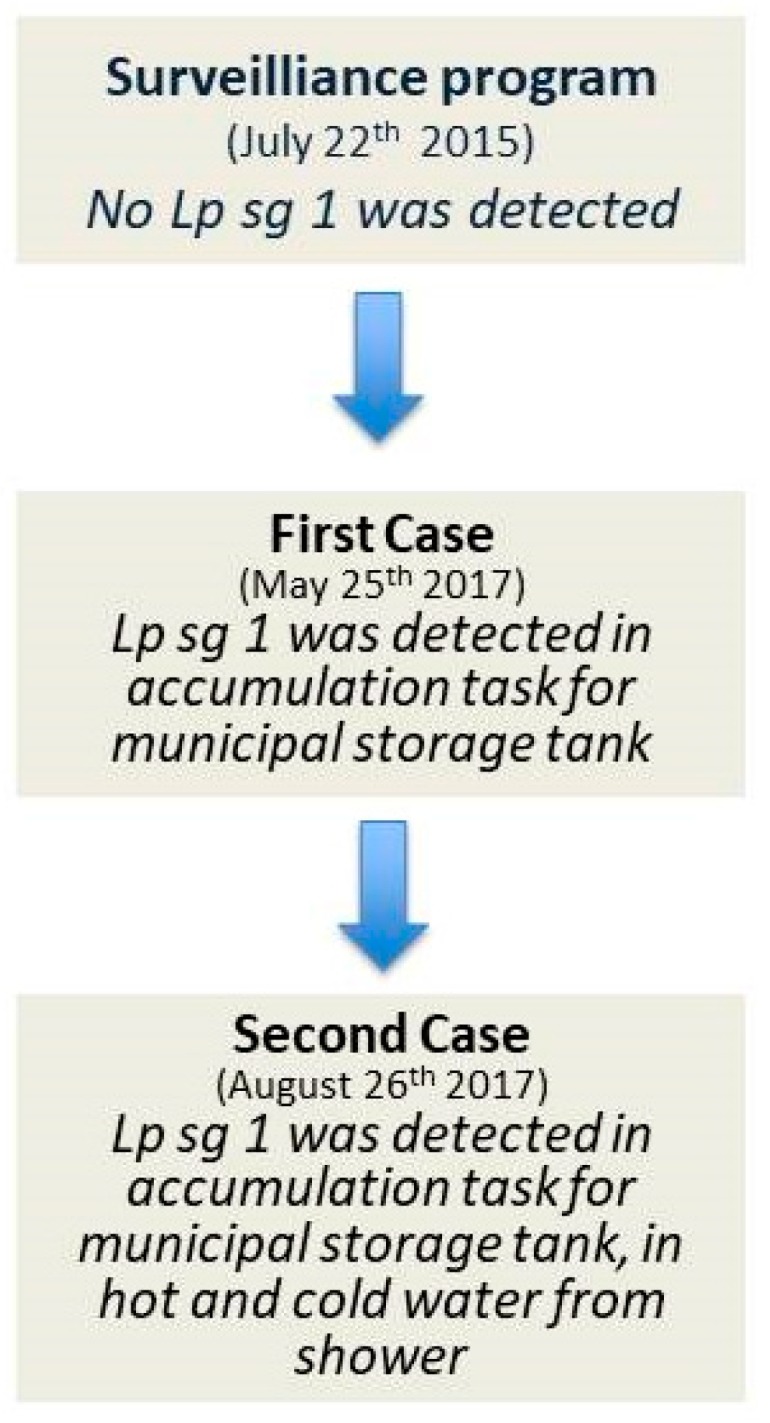
Data regarding a cluster in a Sicilian prison (1PA).

The data are shown in Figure 1.

## 4. Discussion

Still today, water risk management is focused on disinfection and/or maintaining a specific temperature levels in order to control Legionella colonization in the water distribution systems of hospitals and tourist spots. Moreover, recent global epidemiological data show that almost 80% of the cases of LD diagnosed each year are contracted in the civilian community [16,17].

Epidemiological studies reported that Lp sg 1 is predominant in environmental water and is the most frequent serogroup associated with the disease, followed by Lp sg 6 [5].

Our study showed that Lp sg 6 was the most prevalent serogroup in the water samples from Sicilian prisons and confirmed that Lp sg 1 was the only serogroup responsible for the cluster in one Palermo prison. Despite the moderate presence of Lp in environmental water samples, the two prisoners with LD showed comorbid behaviour (alcohol and tobacco consummation). Molecular typing provided very interesting and complete information that helped establish the type of Lp that was circulating, define the interrelationships between clinical and environmental strains, and identify a new ST, that had never before been isolated or typed in Europe or any extra-European country. This new profile was included in the ESGLI-SBT database as the ST 2451, and differed from ST 1362 only in its *neuA* allele.

Molecular typing methods for discriminating between different bacterial isolates are essential epidemiological tools for the prevention and control of Legionella infections and outbreaks.

On the basis of our findings, the Laboratory of Public Health communicated with the prisons and initiated infection-control measures in order to develop Legionella risk management strategies. We ensured that physicians were aware of possible Legionella colonization in the prison water supply and advised them to recommend Legionella diagnostic testing for patients with suspected pneumonia.

Although Italy’s guidelines for the prevention and control of Legionellosis were updated in May 2015, the new document does not take into account prisoners at risk of Legionellosis. It would be desirable to include this population in the document.

Additionally, diagnostic tools that evaluate non-serogroup 1 Legionella antigen in urine should be used in hospitals to investigate other emerging serogroups.

## 5. Conclusions

In Italy and worldwide time-series, analysis of LD incidence demonstrated an increasing incidence of the disease, making LD an important cause of mortality, particularly for immunocompromised patients [13]. Thus, the incidence of LD is rising, and the mortality rate remains high. Any water system producing aerosols can be contaminated by *Legionella* spp. Therefore, the risk of these systems transmitting *Legionella* spp. (or the opportunistic bacteria) should be taken into account, in prisons as well as elsewhere.

Molecular typing is important to understand the evolving response of pathogens to environment changes when disinfection procedures are undertaken and when evaluating the circulation of certain isolates that could be involved in clusters.

In our investigation, by means of molecular typing, we found that prison water systems were clearly implicated as the source of the cluster.

Finally, we recommend a complete and constant monitoring of water systems in prisons because penitentiary populations contain vulnerable people.

## Figures and Tables

**Table 1:** Level of *Legionella pneumophila* (Lp) serogroup (sg) 1 and sg 6 in samples analyzed before the cluster.

Prison	Number of Samples	% of Lp sg 1				% of Lp sg 6				% of Lp <100 CFU/L
		100 CFU/L	101–1000 CFU/L	1001–10,000 CFU/L	>10,000 CFU/L	100 CFU/L	101–1000 CFU/L	1001–10,000 CFU/L	>10,000 CFU/L	
1PA	10	-	-	-	-	10%	20%	-	-	70%
2PA	9	-	-	-	-	-	11.1%	11.1%	11.1%	66.6%
3PA	11	-	36.4%	18.2%	-	-	9.1%	9.1%	45.4%	27.3%
4PA	11	18.8%	9.1%	-	-	9.1%	-	-	-	63.6%
5TP	9	-	-	-	-	-	22.2%	33.3%	-	44.4%
6TP	9	-	-	-	-	11.1%	22.2%	44.4%	11.1%	11.1%
7TP	20	10%	10%	-	-	10%	10%	5%	5%	60%
8AG	8	12.5%	-	-	-	12.5%	-	37.5%	-	50%
9AG	6	-	16.6%	-	-	-	-	-	-	83.3%
	Total = 93									

Lp: *Legionella pneumophila*; Sg: Serogroup; CFU/L: Colony Forming Unit/Liter; 1PA, 2PA, 3PA, 4PA prisons located in Palermo; 5TP, 6TP, 7TP prisons located in Trapani; 8AG and 9AG prisons located in Agrigento.

**Table 2:** Distribution of *Legionella pneumophila* (Lp) serogroup (sg) 1 and sg 6 in water samples analyzed before the cluster.

Site of Origin	n° of Samples	% of Sample Positive for Lp sg 1		% of Sample Positive for Lp sg 6	
		Cold Water	Hot Water	Cold Water	Hot Water
1PA	10	-	-	30%	-
2PA	9	-	-	22.2%	11.1%
3PA	11	27.27%	27.27%	36.36%	27.27%
4PA	11	9.09%	18.18%	9.09%	-
5TP	9	-	-	44.4%	11.1%
6TP	9	-	-	44.4%	44.4%
7TP	20	10%	10%	5%	25%
8AG	8	-	12.5%	-	50%
9AG	6	-	16.66%	-	-

Lp: *Legionella pneumophila*; Sg: Serogroup; CFU/L: Colony Forming Unit/Liter; 1PA, 2PA, 3PA, 4PA prisons located in Palermo; 5TP, 6TP, 7TP prisons located in Trapani; 8AG and 9AG prisons located in Agrigento.

## Abbreviations

Lp	*Legionella pneumophila*
Sg	Serogroup
CFU/L	Colony Forming Unit/Liter
SBT	Sequence-Based Typing
ESGLI	European Study Group for Legionella Infections
ST	Sequence Type
BCYE	Buffered Charcoal Yeast Extract
LD	Legionnaires’ Disease
BAL	bronchoalveolar lavage
ICT	immunochromatographic

## References

[1] Carratalà J., Garcia-Vidal C. (2010). An update on Legionella. Curr. Opin. Infect. Dis..

[2] Mercante J.W., Winchell J.M. (2015). Current and emerging legionella diagnostics for laboratory and outbreak investigations. Clin. Microbiol. Rev..

[3] Rota M.C., Caporali M.G., Bella A., Ricci M.L., Napoli C. (2013). Legionnaires’ disease in Italy: Results of the epidemiological surveillance from 2000 to 2011. Eurosurveillance.

[4] Ministero della Salute (2015). Linee guida per la Prevenzione ed il Controllo della Legionellosi. http://www.salute.gov.it/imgs/C_17_pubblicazioni_2362_allegato.

[5] Italian National Institute of Health (2016). Rapporto Annuale Sulla Legionellosi in Italia Nel 2015.

[6] Helbig J.H., Bernander S., Etienne J., Gaia V., Lauwers S., Lindsay D., Lück P.C., Marques T., Mentula S., Peeters M.F. (2002). Pan-European study on culture-proven Legionnaires’ disease: Distribution of *Legionella pneumophila* serogroups and monoclonal subgroups. Eur. J. Clin. Microbiol. Infect. Dis..

[7] European Centre for Disease Control and Prevention (ECDC) (2016). Legionnaires’ Disease Surveillance in Europe, 2014.

[8] Annual Reports of Legionellosis. www.iss.it.

[9] Delia S., Laganà P., Minutoli E. (2007). Occurrence of legionella in beach shower facilities. J. Prev. Med. Hyg..

[10] Delia S., Laganà P., Minutoli E., Cannavò G., Parisi S. (2008). Prevention of legionellosis in hotel establishments: A proposal to introduce a plan of action in accordance with Provision 13 January 2005 (Italy). Ig. Sanità Pubbl..

[11] Laganà P., Gambuzza M.E., Delia S. (2017). Prevalence and distribution of Legionella pneumophila in cruise ships and ferries. Ann. Agric. Environ. Med..

[12] Gaia V., Fry N.K., Afshar B., Lück P.C., Meugnier H., Etienne J., Peduzzi R., Harrison T.G. (2005). Consensus sequence-based scheme for epidemiological typing of clinical and environmental isolates of Legionella pneumophila. J. Clin. Microbiol..

[13] Ratzow S., Gaia V., Helbig J.H., Fry N.K., Lück P.C. (2007). Addition of neuA, the gene encoding N-acylneuraminate cytidylyl transferase, increases the discriminatory ability of the consensus sequence-based scheme for typing Legionella pneumophila serogroup 1 strains. J. Clin. Microbiol..

[14] Farhat C., Mentasti M., Jacobs E., Fry N.K., Lück C. (2011). The N-acylneuraminate cytidyltransferase gene, neuA, is heterogenous in Legionella pneumophila strains but can be used as a marker for epidemiological typing in the consensus sequence-based typing scheme. J. Clin. Microbiol..

[15] ESGLI (2015). The ESGLI SBT Database for the Typing of Legionella Pneumophila. http://www.hpa-bioinformatics.org.uk/legionella/legionella_sbt/php/sbt_homepage.php.

[16] European Centre for Disease Prevention and Control (2017). Legionnaires’ Disease–Annual Epidemiological Report for 2015.

[17] Garrison L.E., Kunz J.M., Cooley L.A., Moore M.R., Lucas C., Schrag S., Sarisky J., Whitney C.G. (2016). Vital signs: Deficiencies in environmental control identified in outbreaks of Legionnaires’ disease-North America, 2000–2014. Am. J. Transplant..

